# Quit Experiences among Primary Care Patients Enrolled in a Smoking Cessation Pilot RCT Early in the COVID-19 Pandemic

**DOI:** 10.3390/ijerph18031011

**Published:** 2021-01-24

**Authors:** Andrea A. Joyce, Grace M. Styklunas, Nancy A. Rigotti, Jordan M. Neil, Elyse R. Park, Gina R. Kruse

**Affiliations:** 1 Division of General Internal Medicine, Massachusetts General Hospital, Boston, MA 02114, USA; gstyklunas@mgh.harvard.edu (G.M.S.); nrigotti@partners.org (N.A.R.); 2 Tobacco Research and Treatment Center, Massachusetts General Hospital, Boston, MA 02114, USA; jmneil@mgh.harvard.edu (J.M.N.); epark@mgh.harvard.edu (E.R.P.); 3 Department of Public Health and Professional Degrees, Tufts University School of Medicine, Boston, MA 02111, USA; 4 Department of Medicine, Harvard Medical School, Boston, MA 02115, USA; 5 Health Policy Research Center, Mongan Institute, Department of Medicine, Massachusetts General Hospital, Boston, MA 02114, USA

**Keywords:** nicotine, COVID-19, risk perceptions, smoking cessation, tobacco use

## Abstract

The impact of the COVID-19 pandemic on US adults’ smoking and quitting behaviors is unclear. We explored the impact of COVID-19 on smoking behaviors, risk perceptions, and reactions to text messages during a statewide stay-at-home advisory among primary care patients who were trying to quit. From May–June 2020, we interviewed smokers enrolled in a 12-week, pilot cessation trial providing text messaging and mailed nicotine replacement medication (NCT04020718). Twenty-two individuals (82% white, mean age 55 years), representing 88% of trial participants during the stay-at-home advisory, completed exit interviews; four (18%) of them reported abstinence. Interviews were thematically analyzed by two coders. COVID-19-induced environmental changes had mixed effects, facilitating quitting for some and impeding quitting for others. While stress increased for many, those who quit found ways to cope with stress. Generally, participants felt at risk for COVID-19 complications but not at increased risk of becoming infected. Reactions to COVID-19 and quitting behaviors differed across age groups, older participants reported difficulties coping with isolation (e.g., feeling disappointed when a text message came from the study and not a live person). Findings suggest that cessation interventions addressing stress and boredom are needed during COVID-19, while smokers experiencing isolation may benefit from live-person supports.

## 1. Introduction

There is growing concern surrounding the impact of smoking on susceptibility and severity of coronavirus disease (COVID-19). Respiratory symptoms caused by cigarette use, along with the behavioral hand-to-mouth act of smoking, may increase smokers’ risk of infection for COVID-19 [1,2]. To date, several studies have found that smoking is a risk factor for severe disease among hospitalized patients with COVID-19 [3,4]. COVID-19 mortality rates are also higher among smokers [5,6,7]. Furthermore, there is evidence that smoking-related conditions, including cardiovascular disease, COPD, and diabetes mellitus, are also associated with poorer COVID-19 outcomes [8,9,10,11,12]. The World Health Organization has recommended that smokers take immediate action to quit to reduce their risk of infection and severe COVID-19 outcomes [13]. As the pandemic continues, it remains essential to focus on prevention practices. Given the role of cigarettes in the disease progression of COVID-19, tobacco cessation is a public health priority.

While research has emphasized the role of cigarettes in the severity of COVID-19 cases [14], research also suggests that nicotine may reduce the risk of being infected with COVID-19 [4]. It remains unclear how the pandemic affects smokers’ perceived risks of COVID-19 infection and illness severity. Additionally, there is little information available on how US smokers are receiving messages about the effect of smoking on COVID-19 risks and whether the information they are receiving is accurate. It is possible that during isolation, similar to hospitalization, the disruption in routines and heightened vulnerability could promote quitting [15,16]. Alternatively, the uncertainty of financial strain and stress during the pandemic may be a driver of smoking behavior. During the 9/11 tragedy, reported stress elevation led to smoking relapse [17,18]. Similarly, following hurricane Katrina, Louisiana residents reported more distress and depressive symptoms, and these mediated smoking relapse [19,20].

From March 2020, US states began the implementation of stay-at-home advisories in order to slow the spread of the virus [21]. With these measures in place, healthcare systems had to rapidly transition from in-person encounters to remote or virtual care methods [22,23]. To date, digital cessation aids including mobile applications and text message programs have effectively promoted self-efficacy and increased abstinence [24,25,26]. With smoking being one of the main preventable causes of COVID-19 progression, smokers may be more inclined to seek out treatment options at this time. Therefore, it is crucial to understand how patients are responding to digital tobacco cessation interventions [5].

The objective of the current study was to explore the impact of COVID-19 on smoking behaviors and risk perceptions, and to assess reactions to a remote cessation program with text messages and mailed nicotine replacement medications among primary care patients enrolled in a quit smoking trial during the initial surge of the pandemic and a statewide stay-at-home advisory.

## 2. Materials and Methods

### 2.1. Participants

From May to June 2020, qualitative interviews were conducted among participants actively enrolled in an ongoing, 12-week, pilot sequential multiple assignment randomized trial (NCT04020718). Participants were patients in primary care practices in an Eastern Massachusetts healthcare system. Inclusion criteria included: (1) 18 years or older; (2) listed as a current daily smoker in the electronic health record (EHR); (3) preferred language listed as English in EHR; (4) PCP within the healthcare system; (5) visited their PCP within the last two years; and (6) mobile phone number listed in their EHR. We excluded all individuals who were currently pregnant, planning to become pregnant in the next three months or breastfeeding, and those currently taking smoking cessation medications. The trial was registered on ClinicalTrials.gov prior to the COVID-19 pandemic. The trial and qualitative interviews were approved by the healthcare system’s institutional review board. Participants who completed interviews were provided a $20 gift card as remuneration for their time.

### 2.2. Intervention

Participants enrolled in the trial were initially randomized, using a computer-generated random sequence, to early (four weeks) assessment or late (eight weeks) assessment of response to a text message intervention. At assessment timepoints, those still smoking by self-report were randomized a second time to receive either mailed Nicotine Replacement Therapy (NRT) or NRT plus a telephone tobacco cessation coaching session. Regardless of treatment assignment, all participants were sent automated text messages for up to 12 weeks. The text message program content is described elsewhere [27,28].

Due to the stay-at-home advisory, we edited our standard message content to remove messages that encouraged activities inconsistent with social distancing recommendations. We substituted these removed messages with a message acknowledging the increased stress many participants could be facing and encouraging participants’ efforts to improve their health by quitting smoking and a link to self-care tips during COVID-19.

### 2.3. Data Collection Process

Quantitative measures of participant characteristics were collected by telephone or email survey during trial enrollment and 12-weeks post-enrollment. Surveys assessed gender, age, race, and health insurance. Mood symptoms were assessed using the Patient Health Questionnaire- 2 (PHQ-2) for depressive symptoms [29] and the Generalized Anxiety Disorder-2 (GAD-2) for anxiety symptoms [30]. Nicotine dependence was measured via the two-item Heaviness of Smoking Index [31].

Focusing on the qualitative data collection activities reported here, our study methods and results are reported in line with the Consolidated Criteria for Reporting Qualitative Research (COREQ) [32]. All trial participants were invited to complete an exit interview. Interviews were conducted by telephone by the study coordinator, who participated in qualitative interview training with the PI (GK) plus institutional qualitative training courses, which are overseen by study co-investigators (EP, JN). The interviewer (AJ) was also the tobacco coach and the study coordinator for the trial and hence had an established relationship with participants prior to conducting the interviews. The semi-structured interview guide was initially designed to examine participant experiences with intervention components. The guide was revised during initial stay-at-home advisory to explore the impacts of the pandemic. Domains included (1) the impact of changes in the physical environment and social distancing on quitting and relapse, (2) stress and coping, (3) perceived risks of COVID-19 infection and severe outcomes, and (4) experiences with cessation treatments during the stay-at-home advisory. Interview questions were open-ended and lasted 14 min on average. Interviews were conducted by telephone and were recorded and transcribed verbatim.

### 2.4. Qualitative Analysis

Interview transcripts were analyzed using NVivo 12 software (QSR International, Victoria, Australia) and a Framework Analysis approach [33]. A preliminary coding framework was developed based on a priori themes from the interview guide and combined with emergent findings from the raw data. This coding framework was iteratively refined using raw data and discussion among the team with the constant comparative method to enhance validity [34]. The final coding framework was applied to all transcripts using descriptive coding as the first cycle coding method by two coders (AJ and GS) trained in qualitative coding methods [35]. To complement descriptive coding, researchers produced written narratives for each transcript, which provided a brief overview of the participant’s experience. In second cycle coding, coding matrices [35] were used to assess differences in COVID-19 impacts by quit status, gender, age cohorts under 55 (*n* = 11, 50%) versus over 55, psychiatric symptoms and trial group assignment to explore heterogeneity in pandemic impacts on smoking and cessation.

## 3. Results

Of those enrolled in the trial during the stay-at-home advisory, 22 participants (88%) completed exit interviews. Sociodemographic characteristics of participants are outlined in Table A1. The mean age of participants was 55 years (*SD* = 14.4), and four participants (18%) reported abstinence at 12 weeks. At the time of interview, none of our participants had reported testing positive for COVID-19. Our results include five central themes characterizing participants’ experience of quitting during the pandemic.

### 3.1. Theme 1: Effect of COVID-19-Induced Environmental Changes on Smoking Behaviors

Interviewees had varied experiences with COVID-19-induced environmental changes, and these changes had a range of effects on their smoking behaviors.

#### 3.1.1. Negative Effect of Environmental Changes on Smoking Behaviors

Over half of participants mentioned struggling with boredom during their time in the study, mainly attributed to being home. The lack of alternative activities available during the stay-at-home advisory had negative implications for their quit attempts:

*“I think what happened was I was just getting so bored that—when you’re bored, what do you do? You eat, and I smoke. I don’t drink. I don’t do drugs. But that was my out. I was reading 10 books a week, and you get bored, and you get up and you have a cigarette”* [A, under 55, not quit].

Similarly, social distancing measures made cessation more challenging for participants. Some mentioned that being home gave them more freedom to smoke without needing to worry about smoking restrictions in the workplace or out of respect for non-smokers in their company:

*“The coronavirus forced me to stay home from work a lot more than I usually do and not always having a lot to do. Sitting around watching some TV or working around the house outside, I had more freedom, and it was easier to smoke and not be around many people”* [B, under 55, not quit].

#### 3.1.2. Positive Effects of Environmental Changes on Smoking Behaviors

In contrast, staying home had favorable implications on quit attempts for others as they were trying to avoid leaving their homes to go to a store to buy cigarettes. Going out to socialize was no longer permitted, leading to less exposure to triggers such as drinking at a bar. Furthermore, participants reported that wearing a mask while in public prohibited them from smoking. Social distancing measures also helped participants to cut down or quit as not being around other smokers removed the temptation to smoke:

*“Well, if I went out, I would have the mask on so I wouldn’t smoke a cigarette”* [C, under 55, not quit].

*“I would think it would be harder if the coronavirus wasn’t around because. I’m not going out to a pub, not meeting with my friends”* [D, over 55, not quit].

### 3.2. Theme 2: Pandemic-Induced Stress and Smoking

Many participants mentioned experiencing heightened stress due to the fear and the uncertainty of COVID-19, which negatively impacted quitting behaviors. Some began to question whether to even to try a quit attempt at this time:

*“The fact that I’m not working and the pandemic, obviously, didn’t really help because it’s a very stressful time. So I was thinking to myself, ‘Is this the right time to even try (to quit),’ considering that I do have all this added problematic situation and stress”* [E, under 55, quit].

Others who had already committed to quitting mentioned how difficult it was to refrain from smoking while trying to deal with the heightened stress. Most used their cigarettes to cope and were satisfied with reducing their smoking rather than quitting:

*“I think considering the stressful nature of the world right now, I think it was a fairly successful attempt to quit. Just another factor in the pandemic and how it affects me is I work at [grocery store]. And right now, because of the pandemic, work is much more stressful than it is normally, unfortunately. So, I think it’s another reason that made it harder to quit”* [F, under 55, not quit].

Those who reported abstinence during their exit interviews mentioned finding alternative ways to deal with stress, such as exercising, and emphasized their reliance on cessation medications at this time:

*“I’ll walk around that grassy area five times to get the mile in now, and that seems to de-stress me, but I only do that when there’s no people out there. Everybody’s out there trying to stretch, so I just make sure it’s at a time when there’s nobody out there. And then I do the stretching—I learned I forget what it’s called body shape or body stretch or something. It’s not so much to lose weight as it is to just stretch. So I’ll do that in the apartment also to not smoke, but I got to be honest with you and tell you that I really rely on those lozenges”* [G, over 55, quit].

### 3.3. Theme 3: No Increased Risk of Being Infected Due to Smoking

The pattern of risk perceptions was generally consistent among participants in that most perceived no increased risk of being infected with COVID-19 due to their smoking. Participants emphasized that they were following the COVID-19 prevention guidelines and, therefore, believed they had the same risk of being infected as a non-smoker. They believed that infection transmits through exposure to others and is unrelated to smoking:

*“Well, I think it’s transmitted from person to person, not from cigarettes”* [H, over 55, not quit].

Some participants believed that their smoking might even offer protection from being infected:

*“This might sound funny, but I was talking to somebody who runs an area in the hospital when all this first started, and that guy didn’t even know that I smoked, but he said to me and another guy in conversation that—he said, ‘Do you know that smokers are less likely to get coronavirus because their lungs have a buildup in them [laughter]?’ That’s something that come out of somebody’s mouth, and it kind of shocked me. I don’t know where he got the information from, but it was something that stuck with me”* [I, under 55, not quit].

### 3.4. Theme 4: Perceived Increased Risk of Poor Outcomes Due to Smoking If Infected with COVID-19

Despite not seeing any relationship between their smoking and their likelihood of contracting COVID-19, most participants believed they were more vulnerable to COVID-19 infection complications due to the effect of smoking on their lungs. Complications mentioned included increased difficulty recovering from the infection and a greater likelihood of experiencing severe illness, including death, compared to non-smokers. Most participants who endorsed this belief in the negative consequences of smoking on COVID-19 disease outcomes reported that this risk perception did not influence their motivation to quit. However, two participants who quit reported that the increased risk of infection complications motivated them to do so:

*“Did the coronavirus make you want to quit more or less or no change?”* [Interviewer].

*“It definitely did because it’s a respiratory infection. And people that are longtime smokers or have asthma are more prone to have more crippling sicknesses if they do get it. So I think that that was in the back of my mind. I mean, the coronavirus is scary. I don’t care what anyone says. It’s really scary. If you are young and you have no high risk, then I can see where it would be less scary if you get it. You can get through it at home, and then you’ll be fine afterwards. And if you’re more high risk, it’s kind of roulette. You don’t know what’s going to happen”* [E, under 55, quit].

### 3.5. Theme 5: Different Patterns of Impacts of COVID-19 on Smoking Behaviors

The impact of COVID-19 on smoking behaviors did not appear to differ significantly by quit status, psychiatric symptoms measured using the PHQ-2 and GAD-2, nicotine dependence or treatment groups. However, patterns in COVID-19 impact themes did vary by age group (Table A2).

Younger participants aged 25 to 54 years mentioned experiencing increased stress at this time, while increased stress was not often described by our older participants. Older participants aged 55 to 85 years did, however, more often report struggling to deal with social distancing measures. Compared to younger participants, they expressed more difficulties coping with boredom and feelings of isolation, which negatively impacted their quit attempt.

#### 3.5.1. Age and Response to COVID-19 Influenced Reactions to the Texts

There were also different patterns in reaction to the text messages. Most of the participants under 55 years, and all of the participants who reported abstinence, enjoyed the words of encouragement and the social support provided by the messages at this time:

*“They were just amazingly uplifting to me in my life in trying to quit, more than just relying on friends and family and so on and so forth”* [J, under 55, quit].

A minority of the older and younger participants mentioned that the program under different circumstances might have been helpful just not during the context of the pandemic. Although we removed message content that was inconsistent with social distancing measures and added resources on self-care during the pandemic, older participants mentioned that they would have liked the message content to address the fact that they were all “*going through a very difficult time*” [K, over 55, not quit]. For some of our over 55 interviewees, receiving constant automated messages from a machine rather than a loved one or a healthcare provider during isolation caused disappointment:

*“I have to be isolated because of my cancer and heart problems, so every time that the bell went off that I was receiving a text message, I should tell you the truth, I honestly thought it was someone that cared for me in my family or a doctor or something like that, and it got to be almost moronic”* [L, over 55, not quit].

#### 3.5.2. Age Differences in Quitting Behaviors during COVID-19

Younger participants more often reported increased motivation to make a quit attempt due to perceived risks of poor COVID-19 outcomes compared to our older participants. A subset of participants, both older and younger, reported a negative effect of COVID-19 on their desire to quit; however, older participants were more likely to report that COVID-19 had no impact on their desire to quit. Although they experienced more stress, there were more younger participants who reported abstinence at 12 weeks. Overall, younger participants more often reported an optimistic outlook as result of not being around other smokers and considered it an opportunity to quit and to be healthier:

*“I say the coronavirus gave me the opportunity to protect myself from two different health things. One, from coronavirus, and one, to want to live long and live healthy. It gave me time to sit and meditate and self-reflect”* [M, under 55, quit].

## 4. Discussion

Our study findings provide insight into the experiences of participants trying to quit smoking during a stay-at-home advisory prompted by the COVID-19 pandemic. Social distancing and changes in the physical environment had varied implications for cessation attempts in our sample. While protective measures against COVID-19, such as wearing a mask in public, assisted some in reducing their smoking, other factors such as restriction in movement, boredom, and stress during the stay-at-home advisory impeded quit attempts for others. Although several participants mentioned that pandemic-induced environmental changes aided their cessation attempt due to a decrease in opportunities for social smoking, being home more, and not having to comply with smoking restrictions commonly practiced in the workplace led others to increase their smoking. These results suggest that encouraging people who smoke to adopt smoke-free policies for their homes may support their cessation efforts during the pandemic.

Consistent with quantitative research findings in both the US [15] and Australia [36], our results highlighted a link between increased stress and smoking during the pandemic. Participants’ experiences also supported the idea that stress may have different effects on tobacco cessation for different people [37]. While many participants reported using their smoking as a coping mechanism, a few reported that stress due to heightened risk perceptions stimulated them to make a quit attempt [15]. Although stress affected quit intentions in our participants, boredom was highlighted as a more impactful barrier to cessation. This aligns with prior research describing smokers becoming overwhelmed with boredom due to being confined to their homes and having reduced social interactions [38] and boredom playing a crucial role in predicting cessation outcomes [39,40]. The confines of lockdown have the potential for harmful behavior such as increased alcohol consumption [41] in some and self-improvement behavior in others. Indeed, one participant viewed this period of confinement as the perfect opportunity to make a quit attempt. However, this topic did not emerge as a central theme within our analysis and it remains unclear what promoted various reactions to the stay-at-home advisory. These results have implications for cessation practices; though it is essential smokers are aware of their risk at this time, risk perceptions rarely drove cessation practices in our sample. It may be more beneficial to implement supports systems to help smokers deal with stress and boredom rather than emphasizing their health risks.

Our interviews provided insight into the risk perceptions of smokers who were already trying to quit smoking during the initial surge of COVID-19. The majority of study participants did not perceive themselves at increased risk of contracting COVID-19. However, consistent with other research to date, most participants were aware of their risk of severe consequences, including death, if they were to be infected [42]. In our sample, risk perceptions were not strongly influencing intentions to quit. A minority of our participants reported more motivation to quit at this time, but few attributed this change in motivation to COVID-19 risks. These findings complement that of Klemperer et al. (2020), indicating that smokers have mixed reactions to COVID-19, with only some experiencing an increase in motivation to quit and others experiencing no effect or even a decrease in desire to quit [43]. These changes in motivation may not equate to greater abstinence in the pandemic context. Studies examining cigarette and cigar smokers’ responses during the pandemic found motivation to quit increased for some, but increased motivation did not predict abstinence [42,44]. Two of our participants believed that smoking could be beneficial in terms of COVID-19 risks. Although recent research suggests that smokers may be less susceptible to COVID-19 infection [45], the World Health Organization continues to urge smokers to make a quit attempt at this time due to the increased risk of experiencing COVID-19 infection complications [46].

Due to the increased use of remote modalities to support cessation at this time, it is crucial to understand how smokers react to these forms of support [47]. The evidence-based text message program supported the cessation efforts of some participants during the pandemic [48]. However, in the context of the pandemic, the text message program was not useful for other participants. Indeed, some of our participants were disappointed to get automated texts when expecting communications from family and friends. Others felt the messages were not attuned to the circumstances of the pandemic, despite our inclusion of messages acknowledging the heightened stress and encouraging self-care. While there is limited literature on adverse effects associated with digital care [49,50], the disappointment expressed by one of our participants may represent psychological distress in the context of the social isolation imposed by the stay-at-home advisory. Smokers struggling with isolation during the pandemic may be better served by quitlines, which provide behavioral supports through live telephone interactions rather than using automated forms of cessation assistance.

Although our findings are limited by our small sample size, our results suggested different reactions to COVID-19 among age groups. Younger adults reported more stress and also described more motivation to stop smoking due to COVID-19 compared to older adults. These results are somewhat surprising as older adults are at increased risk for more severe COVID-19 outcomes [51]. Increased stress was less often described as influencing the smoking trajectory of older adults compared to younger adults. Perhaps this relates to the strength and vulnerability integrations model (SAVI) [52], which states that older adults develop strength throughout the lifespan by coping with life experience and building skills that help to cope with stress. Alternatively, older participants had a more challenging time coping with boredom, caused mainly by distancing themselves from others, which negatively affected their cessation efforts. Older participants may have been more distanced from their families and with fewer social interactions from activities, such as going to a store, thus causing boredom and isolation [53]. The text message intervention was not a good fit for some of our older participants at this time. It is not yet clear whether the text messaging intervention was not suitable in the context of the pandemic or, more generally, not suited to the needs of older smokers. Regardless of this distinction, providers should consider alternatives to automated cessation interventions among older individuals, similar to those struggling with isolation, such as telehealth appointments, telephone counseling through quitlines or face to face encounters when safe to do so.

### Limitations

Our study was not without limitations. Interviews were conducted by (AJ) who provided tobacco cessation coaching for some participants during the trial and this may have led to more favorable reporting of study components. Our participants were participating in a smoking cessation trial during the initial surge of the pandemic in a state hard-hit early in 2020 and therefore our findings reflect a single site at a single stage of the prolonged pandemic. Our sample size was limited by our trial sample size and while we reached saturation on some themes, others, such as the reported belief that smoking may reduce COVID-19 risks, would benefit from additional interviews with individuals holding this belief. Similarly, our age cohort subgroups were of small sample size and further data collection is needed for a more comprehensive understanding of differences in pandemic response by age. Our comparison of patterns of beliefs and behaviors by smoking status was limited by the small number of participants achieving cessation during the study. Finally, all interviews were conducted while the stay-at-home advisory was still in place and did not examine the long-term impact of COVID-19 on smoking and quitting behaviors.

## 5. Conclusions

This qualitative study revealed that primary care patients who smoke had varied reactions to COVID-19-induced environmental changes and, this had both positive and negative implications for their cessation attempts. Awareness of the risks of smoking on COVID-19 outcomes may drive quit intentions for some, but both stress and boredom were influential barriers to quitting during the stay-at-home advisory. Cessation interventions that address stress and boredom may be more impactful than those highlighting the risks of smokers during COVID-19. Our smoking cessation text message program produced mixed reactions during this time. Some found it helpful, while others found the automated messages disappointing when expecting to hear from loved ones. Cessation supports offering live person interaction such as quitlines, which are free and readily accessible, might be a more acceptable and effective intervention for older individuals and those experiencing social isolation during a pandemic.

## Data Availability

Not applicable.

## Appendix A

**Table A1:** Participant Characteristics.

Participant Sample N = 22	
	*n* (%)
Gender	
Female	12 (54.5)
Age	
Years—Mean [SD]	54.7 [14.4]
Under 55	11 (50)
55 and over	11 (50)
Race	
American Indian, Alaska native	1 (4)
African American	2 (9.1)
White	18 (81.8)
Other	1 (4.5)
Insurance	
Medicare	4 (18.2)
Medicaid/Masshealth	13 (59.1)
Employer	7 (31.8)
Other state or local	1 (4.5)
Individual	1 (4.5)
Mental Health	
PHQ-2 ≥ 3 ^1^	4 (18.2)
GAD-2 ≥ 3 ^2^	11 (50)
Nicotine Dependence	
Low Nicotine Dependence ≤ 2 ^3^	14 (63.6)
Moderate Nicotine Dependence ≤4 ^3^	7 (31.8)
High Nicotine Dependence >5 ^3^	1 (4.5)

^1^ Patient Health Questionnaire−2 item scale. ^2^ General Anxiety Disorder 2-item scale. ^3^ Heaviness of smoking index.

**Table A2:** Patterns of COVID-19 impact by age, comparing participants over and under 55 years.

Subthemes	Summary of Theme	Supporting Quotation
Differences in impacts of COVID-19 on Smoking	Younger participants experienced more stress during this time which made it harder for them to quit.	“*So quitting smoking is stressful. And then I had extra stress added on to try to keep my family safe and me safe, and I think that was definitely the issue. I think if the virus wasn’t here, and I had the text messaging, I definitely would have stopped because I stopped for longer period of time than I ever. So I think it was just the timing that it involved*” [A, under 55, not quit]. *“I don’t know if it’s just stress with everything that’s going. If these, I think, other outside factors weren’t involved, it might have gone a little better for me”* [M, under 55, not quit].
	Older participants struggled with boredom and isolation which led them to smoke more.	“*I think having to stay at home affects it. Social distancing really doesn’t…Because it’s boring. Nothing to do and you have a smoke*” [H, over 55, not quit]. “*Oh, well, because I’m sitting here, and I’m bored, so it’s affected it a lot because, like I said, I can’t keep myself busy enough When I quit drinking, it was the same thing. I had to find things to do to break up your normal thing you would do that would make you want to have a drink. It’s the same thing with cigarettes. You want to change things so that you can quit. When you can’t, well, it’s making it harder*” [N, over 55, not quit].
Age and response to COVID-19 influenced reactions to the texts.	Older participants who were struggling with COVID-19 environmental changes did not enjoy the text messages.	“*I think one of the things that I did sort of feel about it, was the text messages in the last month or so were more of a kind of you-failed-every-day sort of thing. Like, “Did you use the patch or the gum?” No. No. No. And it was more like fail, fail, fail, which, in context of everything else that’s going on, was kind of a bummer*” [O, over 55, not quit]. *“I think the texting was an interesting idea. But, obviously, they were not done that were appropriate for the time what we’re all going through with the COVID, and it was not a person doing it, it was a machine doing it*” [J, over 55, not quit].
	Younger participants more often had a positive reaction to the text messages.	*“From the text messages, I got used to receiving them every day, very much so. They got me very encouraged. It made me feel very empowered as a person to want to quit smoking. And that was one of the things that I was so excited about, to receive text messages. Maybe it wasn’t a call from somebody, but the text messages were very empowering, very strong, and very wonderful to hear and see each morning on different things and different ways that I can use on quitting smoking”* [I, under 55, quit].
Age differences in quitting behaviors during COVID-19	Older participants more often mentioned that COVID-19 had no impact of their motivation to quit.	“*It made no difference whatsoever”* [G, over 55, quit]. *“No, they are pretty much the same benefits* [of quitting]” [O, over 55, not quit].
	Younger participant had a more positive outlook and were more motivated to quit	“*I just kind of figured I’m in my house. I’m not going out. I’m trying to save money. I almost felt like it would be easier in some sense to actually quit now”* [E, under 55, quit].
